# Additional Responsibility for Hospital Managers

**Published:** 1916-04-08

**Authors:** 


### ADDITIONAL RESPONSIBILITY FOR HOSPITAL MANAGERS.

By AN EXPERIENCED LAY SUPERINTENDENT.

As The Hospital has frequently pointed out, the war has added enormously to the responsibilities of hospital managers. The increased cost of provisions, coal, drugs, dressings, linen, washing materials, etc., has added thousands of pounds to the cost of maintenance of institutions. Increased prices are the indirect results of war conditions. Direct causes of new and unexpected expenditure arise from the necessity of insurance. Hospital committees, regarding themselves as trustees of the buildings, have, at considerable outlay, probably and certainly wisely, insured against damage by aircraft under the Government scheme. The recent raids by enemy aircraft have, however, indicated another responsibility, that of making provision for staffs who, in the discharge of duty, may be injured or maimed as the result of aerial attacks. It is generally held that policies of insurance under the Employers' Liability Act or under accident policies do not include war risks. It may be taken for granted they do not, although we know of no case in which liability has been tested. A case which has some bearing upon the point was recently argued before Mr. Justice Bailhache, in which the widow of a passenger on the *Lusitania* brought an action against an insurance company on the ground that death was due to an accident within the meaning of the policy. It was, however, held that war risks were not included, and the action failed.

The immediate point we now urge for consideration is the liability of hospital managers for injuries arising among members of staffs as the result of aerial raids. It may at first hand be held that the question of financial responsibility does not arise. Legally it may not, but the question of moral responsibility does arise and needs careful consideration. All institutions have probably adopted definite rules for their staffs in the event of contingencies arising. Doctors will render assistance to injured members of the public, nurses will be required at once to take up duty in their wards, the staff generally are required to render assistance as circumstances may dictate. In the event of injuries by aerial craft in the performance of such specially imposed duties as these, more likely than not in their off-duty times, the members of hospital staffs and their families may not unreasonably seek to place responsibility upon hospital managers. The advisability of insurance against these risks therefore arises. Inquiries show that only a few insurance companies have issued policies to cover. One well-known company (the Gresham) will, for a yearly premium of 4s., issue policies covering Zeppelin and other aircraft risks only against fatality for £200, loss of two limbs or eyes £200, one limb or eye £100, and temporary incapacity for £1 weekly for fifty-two weeks. Larger or possibly lesser risks would be covered by proportionate premiums. It would certainly seem wise for hospital committees to insure, and the knowledge that they had done so would add to the goodwill of their staffs in readily undertaking additional duties. The moral responsibility is a definite one, and deferment cannot but be without risk. We should welcome the opinion of hospital managers on this new responsibility.

